# Euphrasia Eye Drops in Preterm Neonates With Ocular Discharge: A Randomized Double-Blind Placebo-Controlled Trial

**DOI:** 10.3389/fped.2020.00449

**Published:** 2020-08-11

**Authors:** Delphine Meier-Girard, Gisa Gerstenberg, Liliane Stoffel, Therese Kohler, Sabine D. Klein, Marco Eschenmoser, Vera Ruth Mitter, Mathias Nelle, Ursula Wolf

**Affiliations:** ^1^Anthroposophically Extended Medicine, Institute of Complementary and Integrative Medicine, University of Bern, Bern, Switzerland; ^2^Department of Neonatology, Inselspital, Bern University Hospital, University of Bern, Bern, Switzerland; ^3^Department of Pharmacy, Hospital of Freiburg, Freiburg, Switzerland; ^4^Department of Obstetrics and Gynecology, Inselspital, Bern University Hospital, University of Bern, Bern, Switzerland; ^5^Neonatology Division, University Hospital of Zurich, Zurich, Switzerland

**Keywords:** ocular discharge, congenital nasolacrimal duct obstruction, preterm neonate, Euphrasia drops, complementary medicine

## Abstract

**Aim:** To investigate whether the early administration of Euphrasia eye drops® in preterm neonates presenting with ocular discharge fosters the resolution of the ocular discharge and reduces the need for topical antibiotic therapy, as compared to placebo.

**Methods:** We conducted a randomized double-blind placebo-controlled trial at the University Children's Hospital Bern, Switzerland. Preterm neonates with white, yellow, or green ocular discharge were included. Infants were randomly assigned (1:1) to the Euphrasia arm (Euphrasia eye drops®, Weleda AG, Arlesheim) or the placebo arm (NaCl 0.9%). Euphrasia or placebo was administrated at a dose of one drop in each eye four times a day over a period of 96 h. The primary outcome was the treatment success, defined as no ocular discharge at 96 h and no use of topical antibiotic therapy during the 96-h intervention.

**Results:** A total of 114 neonates were screened and 84 were randomized. Among neonates in the Euphrasia arm, 22 (55.0%) achieved our primary outcome compared to 21 (51.2%) in the placebo arm (*p* = 0.85). In the Euphrasia arm, time to resolution of reddening tended to fall within the shorter bracket of 24 to 48 h (24 (92.3%) vs. 12 (80.0%) in the placebo arm, *p* = 0.34) and relapse or first signs of reddening during the 96-h intervention tended to be lower [3 (7.9%) eyes vs. 8 (18.2%) eyes in the placebo arm, *p* = 0.17]. Tearing at 96 h tended to be lower in the Euphrasia arm [5 (12.8%) eyes in the Euphrasia arm vs. 12 (27.3%) eyes in the placebo arm, *p* = 0.10].

**Discussion:** Euphrasia did not significantly improve treatment success, defined as no ocular discharge at 96 h and no use of topical antibiotic therapy during the 96-h intervention. However, results suggest that Euphrasia may be of benefit for symptoms such as reddening and tearing, and thus improve the comfort of patients.

**Trial Registration:** The trial is registered at the US National Institutes of Health (ClinicalTrials.gov) NCT04122300 and at the portal for human research in Switzerland SNCTP000003490.

## Introduction

Neonatal ocular discharge is mostly related to ophthalmia neonatorum (neonatal conjunctivitis) or to congenital nasolacrimal duct obstruction (CNLDO).

Ophthalmia neonatorum is a relatively common illness, defined as conjunctivitis occurring within the first month of life (1). Ophthalmia neonatorum can be caused by the sexually transmitted pathogens of *Neisseria gonorrhoeae* and *Chlamydia trachomatis*, by bacteria such as *Staphylococcus* species, *Streptococcus* species, *Haemophilus* species and other gram-negative bacterial species, or much less commonly by viral infections (herpes simplex and adenovirus virus). The organisms inducing ophthalmia neonatorum are usually acquired during the birth process, from the mother's birth canal, or after birth from the immediate surroundings (2–4). Epidemiology of organisms that induce ophtalmia neonatorum substantially differs between developing countries and developed countries (3). In the United States *Neisseria gonorrhoeae* counts for <1% of cases and *C. trachomatis* for 2–40% of cases, while 30–50% of cases are caused by other bacteria (1).

Ophthalmia neonatorum must be distinguished from ocular discharge related to CNLDO. CNLDO occurs in ~10–20% of all term newborns, and is the most common cause of persistent tearing and ocular discharge in children. It results from a congenital abnormality of the lacrimal drainage system in the form of a membranous obstruction of the nasolacrimal duct of one or both eyes (5). Spontaneous resolution, through the spontaneous perforation of the membrane, occurs by 6 months of age in ~90% of infants (5). The first clinical signs appear during the first month of life in 95% of cases (5) and usually consist of tearing and debris on the eyelashes (“mattering”). Mucopurulent eye discharge occurs commonly in infants with CNLDO and, in the absence of other signs of infection, suggests bacterial overgrowth in the stagnant tear pool of the lacrimal sac (“chronic dacryocystitis”) (6).

In most instances, ocular discharge is a mild illness. Complication such as microbial keratitis and acute dacryocystitis are rare but can be serious, resulting in severe visual impairment (e.g., corneal diseases), or blindness (7–9). In consequence, topical antibiotic therapy is recommended in case of significant mucopurulent discharge in order to control the bacterial overgrowth, to avoid further inflammatory damage, and to prevent serious infective complications (4, 6, 10, 11). This therapy should be started just after bacterial/viral and chlamydial swabs have been taken. However, the use of antibiotics as the first-line treatment in infants who present with ocular discharge has be questioned in light of other possible treatment strategies (conservative management). At the time of the study it was generally recommended to apply NaCl 0.9% locally in neonates presenting with a white or yellow ocular discharge, and to introduce a topical antibiotic therapy should symptoms become stronger.

A previous pilot study suggested that early treatment with Euphrasia eye drops in 24 neonates with ocular discharge with or without tearing and reddened eye might reduce the antibiotic consumption (12). Euphrasia (eyebright) eye drops (from *Euphrasia officinalis*) are a frequently prescribed medication for the treatment of irritative, infectious, or allergic conjunctivitis and other affections of the eye (13–15). It is also effective against hyperemia. It has been used for more than 70 years for the structuring of the fluid organism in the eye, especially in inflammatory and catarrhal conjunctivitis (13). Euphrasia has an anti-inflammatory effect through the aucubin (inhibition of prostaglandin synthesis), as well as an antibacterial effect through phenolic carboxylic acids and flavonoids. Previous studies have reported excellent tolerance of Euphrasia eye drops in adults and children (12, 13, 15).

The present study—a randomized double-blind placebo-controlled trial—follows our previous pilot study (12) and aimed to investigate whether early administration of Euphrasia eye drops (Weleda AG, Arlesheim) in preterm neonates presenting with ocular discharge with or without tearing and reddened eye fosters the resolution of the ocular discharge and reduces the need for topical antibiotic therapy.

## Methods

### Study Design

We conducted a randomized double-blind placebo-controlled trial between May 2011 and December 2016 at the Department of Neonatology at the Children's University Hospital, Bern, Switzerland. The study was approved by the Ethics Commission of the Canton of Bern, Switzerland (215/08) and written informed consent was obtained from parents or legal guardians of each neonate before any procedures were conducted. The trial was registered at the US National Institutes of Health (ClinicalTrials.gov) NCT04122300 and at the portal for human research in Switzerland SNCTP000003490.

### Study Population

Eligible patients were preterm neonates (with a gestational age of 24 to 37 weeks) diagnosed with white, yellow, or green ocular discharge with or without tearing and reddened eye. The criteria for exclusion were congenital abnormalities of the eye, severe asphyxia, sepsis, or intracranial bleeding (intraventricular hemorrhage ≥ grade III).

### Study Interventions

#### Randomization and Blinding

Infants were randomly assigned (1:1) to receive either Euphrasia or placebo. We used a randomization program (DatInf RandList version 1.0) to generate the randomization lists. Euphrasia or placebo was administrated at a dose of one drop in each eye four times a day over a period of 96 h.

Study investigators, research coordinators, attending care teams and the infants' legual guardian were blinded to treatment allocation. The hospital pharmacy provided the blinded study medication: 0.2 ml of Euphrasia eye drops (Weleda AG, Arlesheim) or 0.2 ml of placebo (sodium chloride 0.9%, Bichsel). The Euphrasia eye drops and placebo were filled in neutral tuberculin syringes of 1 ml under aseptic conditions according to the Good Manufacturing Practices (2010^1^).

#### Treatment Regimen and Microbial Evaluation

At inclusion, before the start of the therapy, a bacterial/viral and chlamydial conjunctival swab was conducted. Afterwards, both eyes of neonates were washed four times a day (i.e., every 6 h) with NaCl 0.9%. Subsequently, a drop of Euphrasia or placebo was placed into the lower conjunctival sac of each eye, and followed by a lacrimal sac digital massage. In case of worsening of symptoms or a positive swab without any improvement of symptoms an antibiotic therapy was initiated: bacitracin, neomycin, polymyxin B (Neosporin ointment, HeliDerm) or tobramycin (Tobrex 0.3% eye drops, Novartis Pharma Schweiz AG) after the Neosporin withdrawal in September 2013. An additional swab was performed at 96 h (i.e., at the end of the study).

### Study Outcomes

The primary outcome was the treatment success, defined as no ocular discharge at 96 h and no use of topical antibiotic therapy during the 96-h intervention period. If a neonate presented a bilateral affection, the therapy was defined as successful only if ocular discharge had disappeared in both eyes at 96 h.

As secondary outcomes, the type of ocular discharge (white, yellow, or green) and the presence of tearing or reddening were recorded at baseline, and at 24, 72, and 96 h, as was the use of topical antibiotic therapy during the 96-h intervention period.

### Sample Size and Statistical Analysis

We calculated that a total of 84 infants would be needed to detect a difference between groups, with a two-tailed α of 0.05 and a power of 80%, for a comparison of two independent proportions if there was an absolute increase of 30% in the primary outcome measure (treatment success). Our initial estimate of sample size calculation included an assumption of treatment success rate of 30% in the placebo arm. Assumptions were based on the results of our pilot study and communication with clinicians (12).

Our primary analysis was conducted applying an intention-to-treat approach, and therefore included all randomized infants. Baseline characteristics of patients in the two treatment groups were reported using frequency distribution and descriptive statistics. Baseline characteristics included demographic characteristics of neonates (i.e., gender, age, and weight), birth characteristics of neonates, and age of mother at birth. Birth characteristics included gestational age at birth (<28 weeks of gestation (WG) was defined as extremely preterm, 28 to 32 WG as very preterm, and 32 to 37 WG as moderate to late preterm, in accordance with the WHO definitions), birthweight and birth procedure (spontaneous birth, instrumental vaginal delivery, or cesarean section).

The principal analysis of our primary outcome was an unadjusted chi-square test comparing the proportion of events in each treatment group.

A per-protocol analysis of infants who filled all inclusion criteria was also conducted to examine the robustness of our primary estimates.

All analyses were conducted with R, Version 3.5.1 (16).

## Results

### Study Population

**Figure 1:**
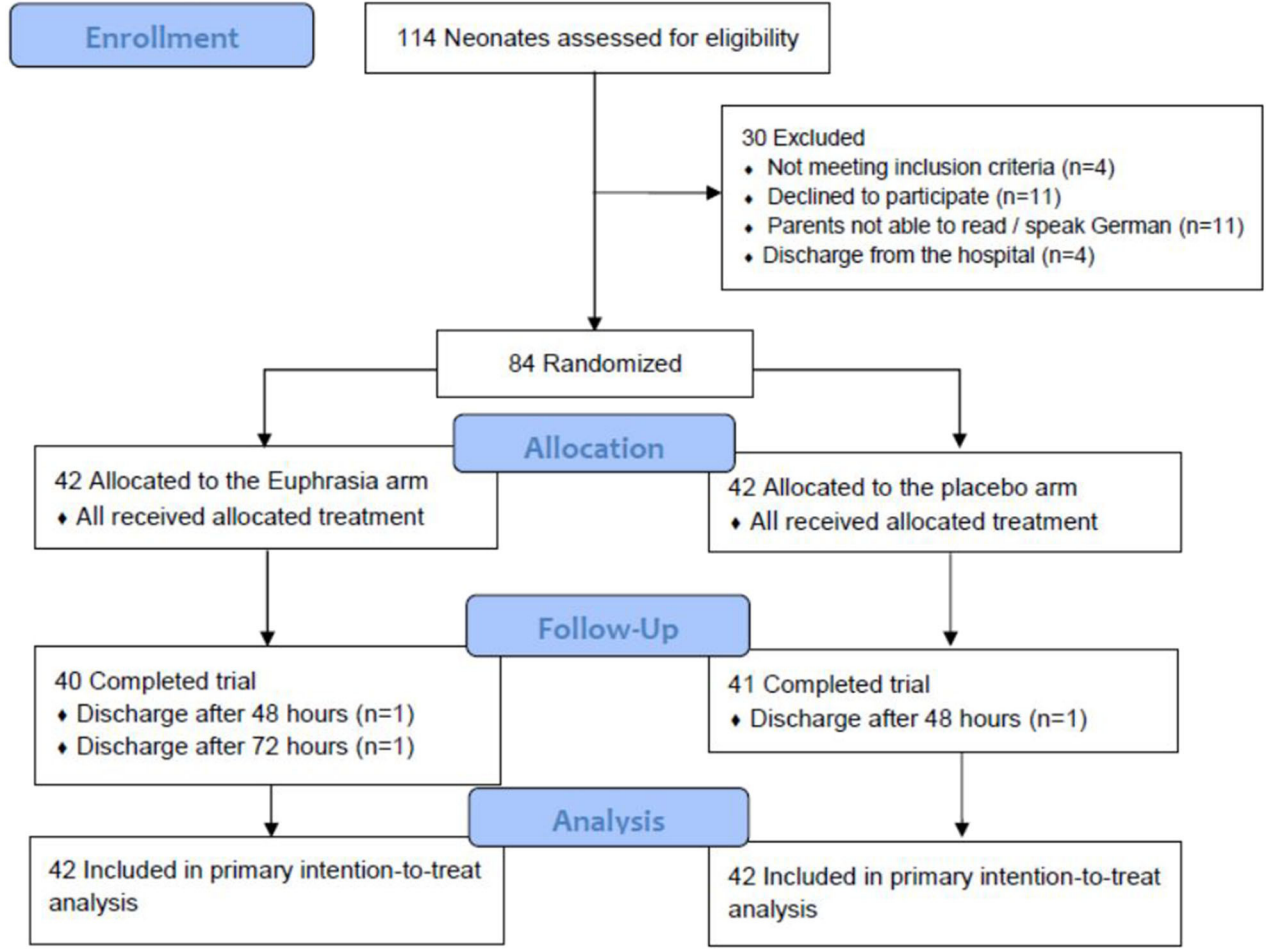
Participant flow.

A total of 114 neonates were screened for eligibility and 84 were randomized between May 2011 and December 2016 into the Euphrasia arm (*n* = 42) and the placebo arm (*n* = 42) (Figure 1). Three neonates were randomized though they did not fill the inclusion criteria (one neonate was born at full-term and two neonates did not present any ocular discharge at inclusion). These three neonates were allocated to the Euphrasia arm. All randomized infants completed the trial with the exception of three infants who were discharged before the end of the study. The primary outcome was consequently missing for these three infants. In total, 48 (60%) were boys, the mean postnatal age was 21 ± 16 days, 42 (52.5%) were moderate to late preterm, 24 (30.0%) were very preterm, and 14 (17.5%) were extremely preterm. Baseline demographic and clinical characteristics were similar in both treatment arms (Table 1).

**Table 1:** Baseline characteristics (*n* = 84 neonates).

	**Euphrasia (*n* = 42 neonates)**	**Missing data**	**Placebo (*n* = 42 neonates)**	**Missing data**
**Demographic characteristics**				
Sex, male	27 (64.3%)	-	24 (57.1%)	-
Postnatal age, days	12 [10–22]	-	17 [12–27]	-
Weight, *g*	1807 ± 523	1	1722 ± 443	-
**Birth characteristics**				
Gestational age at birth, week of gestation^a^		-		-
Extremely preterm <28	7 (16.7%)		8 (19.0%)	
Very preterm [28;31(6/7)]	13 (31.0%)		12 (28.6%)	
Moderate to late preterm [32;36(6/7)]	21 (50.0%)		22 (52.4%)	
Full-term ≥37^b^	1 (2.4%)		0 (0.0%)	
Birthweight, *g*	1,608 [1,226–1,834]		1,498 [988–1,699]	-
Age of mother at delivery, years	35.0 [30.2–37.0]	-	32.5 [29.0–36.7]	-
Birth procedure		-		2
Spontaneous birth	10 (23.8%)		5 (12.5%)	
Instrumental vaginal delivery	2 (4.8%)		3 (7.5%)	
Cesarean section	30 (71.4%)		32 (80.0%)	

*Data are expressed as mean ± standard deviation, median [interquartile range], and number (percentage)*.

a *In accordance with the WHO definitions*.

b *One neonate was randomized though he was born at full-term*.

### Ocular Health at Baseline

Laterality of the ocular discharge (i.e., unilateral or bilateral) was similar in both treatment arms (Table 2). In the Euphrasia arm, 10 (25.0%) neonates presented a bilateral ocular discharge at baseline, resulting in 50 eyes with ocular discharge among the 42 neonates (59.5%). In the placebo arm, 17 (40.5%) neonates presented a bilateral ocular discharge, resulting in 59 eyes with ocular discharge among the 42 neonates (70.2%).

**Table 2:** Laterality of ocular discharge at baseline according to treatment arm.

	**All neonates (*****n*** **=** **84 neonates)**			**No topical antibiotic therapy (*****n*** **=** **68 neonates)**		
	**Euphrasia** ** (*n* = 42 neonates)**	**Placebo** ** (*n* = 42 neonates)**	**MD**	**Euphrasia** ** (*n* = 34 neonates)**	**Placebo** ** (*n* = 34 neonates)**	**MD**
At least one eye with ocular discharge	40 (95.2%)^a^	42 (100.0%)	-	32 (94.1%)^a^	34 (100.0%)	-
Laterality of ocular discharge			-			-
One eye	30 (75.0%)	25 (59.5%)		25 (78.1%)	22 (64.7%)	
Both eyes	10 (25.0%)	17 (40.5%)		7 (21.9%)	12 (35.3%)	

*Data are expressed as number (percentage)*.

a *Two neonates were randomized though they did not present with ocular discharge at baseline*.

Symptoms at baseline were similar in both treatment arms with the exception of the reddening (Table 3). In both treatment arms, neonates presented mainly with yellow ocular discharge, and slight tearing. More neonates presented with green ocular discharge in the Euphrasia arm. In the Euphrasia arm, ocular discharge was accompanied by reddening in more eyes than in the placebo arm. The reddening was bilateral for 5 neonates in the Euphrasia arm, while this was the case for only one neonate in the placebo arm.

**Table 3:** Ocular health at baseline according to treatment arm (*n* = 84 neonates, 111 affected eyes).

	**Euphrasia (*n* = 52 eyes)**	**Missing data**	**Placebo (*n* = 59 eyes)**	**Missing data**
**Symptoms at baseline**				
Ocular discharge	50 (96.2%)^a^	-	59 (100%)	-
Type of ocular discharge		1		-
White	5 (10.2%)		8 (13.6%)	
Yellow	40 (81.6%)		50 (84.7%)	
Green	4 (8.2%)		1 (1.7%)	
Reddening	33 (63.5%)	-	21 (36.2%)	1
Tearing	34 (66.6%)	1	36 (63.2%)	2

*Data are expressed as number (percentage)*.

a *Two neonates were randomized though they did not present with ocular discharge at baseline*.

### Therapy Outcome

#### Primary Outcome

A total of 43 (53.1%) infants met our primary outcome of treatment success. Treatment success did not significantly differ between treatment arms [22 (55.0%) in the Euphrasia arm vs. 21 (51.2%) in the placebo arm, *p* = 0.85] (Table 4).

**Table 4:** Treatment success at 96 h (primary outcome) and use of topical antibiotic therapy according to treatment arm (*n* =8 4 neonates).

	**Euphrasia (*n* = 42 neonates)**	**Missing data**	**Placebo (*n* = 42 neonates)**	**Missing data**	***p*-value^a^**
Success of treatment at 96 h	22 (55.0%)	2	21 (51.2%)	1	0.85
Topical antibiotic therapy	8 (19.0%)	-	8 (19.0%)	-	0.99
Time to initiation of antibiotic therapy^b^		-		-	0.99
Within 48 h	5 (62.5%)		4 (50.0%)		
Between 48 and 96 h	3 (37.5%)		4 (50.0%)		
Decreasing of symptoms after topical antibiotic therapy	6 (75.0%)	-	6 (75.0%)	**-**	0.99

*Data are expressed as number (percentage)*.

*Treatment success was defined as no ocular discharge at 96 h and no use of topical antibiotic therapy during the 96-h intervention period. If a neonate presented a bilateral affection, the treatment was defined as successful only if ocular discharge had disappeared in both eyes at 96 h*.

a *Chi-square test or Fischer test, as appropriate*.

b *In the 8 neonates who received a topical antibiotic therapy*.

#### Topical Antibiotic Therapy

The use of topical antibiotic therapy was similar in both treatment arms (Table 4). Within the 96-h intervention period, 8 (19.0%) neonates in the Euphrasia arm and 8 (19.0%) neonates in the placebo arm received a topical antibiotic therapy (*p* = 0.99). The time to initiation of the antibiotic therapy did not significantly differ between treatment arms and symptoms were observed to decrease similarly in both groups after introduction of topical antibiotic therapy. Neonates who received topical antibiotic therapy presented at inclusion significantly more tearing as compared to neonates who did not receive topical antibiotic therapy [21 (87.5%) eyes with tearing vs. 49 (58.3%) eyes with tearing, respectively, *p* = 0.008] (Table E1).

Among the 16 neonates who received a topical antibiotic therapy, results of swabs performed at inclusion were similar in both groups (Table E2). Swabs were positive for *Staphylococcus aureus (S. aureus)* in 9 neonates. Swabs were negative at inclusion in three neonates. Due to a substantial worsening of symptoms in these three neonates, it had not been possible to wait for the results of the swabs before initiating the antibiotic therapy.

Among the 68 (81.0%) neonates who did not receive any topical antibiotic therapy, results of swabs performed at inclusion were similar in both groups (Table E2). Swabs were positive in 31 (45.6%) neonates [16 (47.1%) in the Euphrasia arm vs. 15 (44.1%) in the placebo arm, *p* = 0.81]. The swabs were mainly positive to *S. aureus* [16/31 (51.2%) swabs]. At 96 h, 21 (70.0%) of the neonates with a positive swab at inclusion and 22 (61.1%) of the neonates with a negative swab at inclusion were free of ocular discharge (*p* = 0.45) (Table E3). Therefore, no significant difference in the success of treatment at 96 h was observed based on the presence of microbiological agents at the beginning of the affection. No significant difference was observed between treatment arms (Table E3).

None of the neonates tested positive for *Neisseria gonorrhoeae* and *C. trachomatis*.

#### Resolution of Ocular Discharge and Evolution of Other Symptoms

Among the 68 (81.0%) neonates who did not receive any topical antibiotic therapy, in the Euphrasia arm, 7 (21.9%) neonates presented a bilateral ocular discharge at baseline, resulting in 39 eyes with ocular discharge among 34 neonates (57.4%) (Table 2). In the placebo arm, 12 (35.3%) neonates presented a bilateral ocular discharge, resulting in 46 eyes with ocular discharge among the 34 neonates (67.6%). Table 5 provides an overview according to treatment arm of how symptoms (i.e., ocular discharge, reddening, tearing) evolved in neonates who did not receive any topical antibiotic therapy over the 96 h of intervention.

- Resolution of ocular discharge was observed in 34 (89.5%) eyes in the Euphrasia arm and in 39 (86.7%) in the placebo arm (*p* = 0.83). Time to resolution of ocular discharge did not differ between arms (*p* = 0.16). Relapse of ocular discharge was significantly higher in the Euphrasia arm as compared to the placebo arm [13 (38.2%) eyes vs. 6 (15.8%) eyes, *p* = 0.03]. Resolution at 96 h was similar in both treatment arms [ocular discharge in 12 (30.8%) eyes in the Euphrasia arm vs. 12 (27.7%) eyes in the placebo arm, *p* = 0.73].
- Resolution of reddening was observed in 26 (96.3%) eyes in the Euphrasia arm and in 15 (100.0%) eyes in the placebo arm (*p* = 0.99). In the Euphrasia arm, time to resolution tended to be shorter [24 (92.3%) vs. 12 (80.0%) in the placebo arm, *p* = 0.34] and relapse or first signs of reddening during the 96-h intervention tended to be lower [3 (7.9%) eyes vs. 8 (18.2%) eyes in the placebo arm, *p* = 0.17]. Reddening at 96 h was similar in both treatment arms [reddening in 1 (2.6%) eyes in the Euphrasia arm vs. 2 (4.5%) eyes in the placebo arm, *p* = 0.99].
- Resolution of tearing was observed in 23 (100.0%) eyes in the Euphrasia arm and in 24 (96.0%) eyes in the placebo arm (*p* = 0.99). Time to resolution did not differ between arms (*p* = 0.99). Relapse of first signs of tearing during the 96-h intervention was similar in both treatment arms (*p* = 0.73). Tearing at 96 h tended to be lower in the Euphrasia arm [5 (12.8%) eyes in the Euphrasia arm vs. 12 (27.3%) eyes in the placebo arm, *p* = 0.10].

**Table 5:** Evolution of symptoms in neonates who did not go on to receive a topical antibiotic therapy according to treatment arm (*n* = 68 neonates, 87 affected eyes).

	**Euphrasia (*n* = 41 eyes)**	**Missing data**	**Placebo (*n* = 46 eyes)**	**Missing data**	***p*-value^a^**
**Ocular discharge**					
Ocular discharge at baseline	39 (95.1%)^b^	-	46 (100.0%)	-	0.99
Resolution during intervention	34 (89.5%)	1	39 (86.7%)	1	0.83
Time to resolution		-		-	0.16
24–48 h	22 (64.7%)		31 (79.5%)		
72–96 h	12 (35.3%)		8 (20.5%)		
Relapse during intervention	13 (38.2%)	1	6 (15.8%)	1	0.03
Ocular discharge at 96 h	12 (30.8%)	2	12 (27.7%)	2	0.73
**Reddening**					
Reddening at baseline	27 (65.9%)	-	15 (33.3%)	1	0.003
Resolution during intervention	26 (96.3%)	-	15 (100.0%)	-	0.99
Time to resolution		-		-	0.34
24–48 h	24 (92.3%)		12 (80.0%)		
72–96 h	2 (7.7%)		3 (20.0%)		
Relapse or first signs during intervention	3 (7.9%)	3	8 (18.2%)	2	0.17
Reddening at 96 h	1 (2.6%)	2	2 (4.5%)	2	0.99
**Tearing**					
Tearing at baseline	24 (60.0%)	1	25 (56.8%)	2	0.77
Resolution during intervention	23 (100.0%)	1	24 (96.0%)	-	0.99
Time to resolution		-		-	0.99
24–48 h	19 (82.6%)		19 (79.2%)		
72–96 h	4 (17.4%)		5 (20.8%)		
Relapse or first signs during intervention	18 (46.2%)	2	22 (50.0%)	2	0.73
Tearing at 96 h	5 (12.8%)	2	12 (27.3%)	2	0.10

*Data are expressed as number (percentage)*.

a *Chi-square test or Fischer test, as appropriate*.

b *Two neonates did not fill the inclusion criteria (intention-to-treat approach)*.

### Sensitivity Analyses

A per-protocol analysis of infants who met all inclusion criteria did not alter the conclusions on the primary (treatment success was 19 (52.8%) in the Euphrasia arm vs. 21 (51.2%), *p* = 0.89) and secondary outcomes (results not shown).

The analysis of the treatment success according to treatment arm and preterm status (extremely and very preterm vs. moderate to late preterm and full-term) did not show any significant difference (results not shown).

## Discussion

### Main Results

In this study with 84 preterm neonates with ocular discharge treated with either Euphrasia eye drops or placebo, Euphrasia did not significantly improve treatment success as compared to the placebo arm, defined as no ocular discharge at 96 h and no use of topical antibiotic therapy during the 96-h intervention.

However, this study shows a remarkably high treatment success rate at 96 h [i.e., no ocular discharge at 96 h and no use of topical antibiotic therapy: *n* = 43 (53.1%)] and a low rate of topical antibiotic use [*n* = 16 (19.0%)]. At the time of the study, treatment success was expected in 3 to 5 days, with 50 to 80% of neonates receiving a topical antibiotic therapy. Treatment success and use of topical antibiotic therapy were similar in both treatment arms. We explain these results by the systematic eye washing and lacrimal sac massage, newly introduced in the Department of Neonatology at the University Children's Hospital, Bern, Switzerland, as part of this study. The massage of the lacrimal sac has been recognized as a very efficient therapy (17), and is recommended as the first-line treatment in CNLDO (18, 19).

We observed a high rate of treatment success at 96 h in neonates with a positive bacteriological swab at inclusion who did not go on to receive a topical antibiotic therapy. No relationship was observed between the microbiological agent identified and the success of treatment. These results support that topical antibiotics should not be used systematically as the first-line treatment of preterm neonates presenting with ocular discharge. Rather, the first-line treatment of lacrimal sac massage should be applied first.

In the Euphrasia arm, a trend was observed for shorter time to reddening resolution as well as lower rate of relapse, suggesting that Euphrasia might be of benefit against reddening. However, the difference was not statistically significant, possibly due to a lack of power.

The rate of relapse of ocular discharge in the subgroup of neonates who did not go on to receive a topical antibiotic therapy was significantly higher in the Euphrasia arm. Some of the relapses in the Euphrasia arm were observed in neonates initially presenting a green ocular discharge, indicating a more severe affection, while no green ocular discharge was observed in the placebo arm. Moreover, in this subgroup, significantly more infants presented with a reddening at baseline in the Euphrasia arm, also indicating a potentially more severe affection. At 96 h, the ocular discharge relapses had resolved in the Euphrasia arm such that there was no significant difference between treatment arms.

Finally, the pattern of tearing fluctuated during the intervention period with high rates of resolution and relapse. This is to be expected given the nature of the mechanical mechanism underlying this symptom. At the end of the intervention, we observed a trend for less tearing in the Euphrasia arm.

### Strengths and Weaknesses of the Study

To the best of our knowledge, this is the first double-blind randomized controlled trial comparing Euphrasia eye drops to placebo in the first-line treatment of ocular discharge. Also, this trial investigated preterm neonates, a vulnerable population that is highly under-represented in clinical trials.

A limitation of the study is the absence of grading of the symptoms (i.e., ocular discharge, tearing, and reddening). We limited our analysis to the presence or absence of symptoms to avoid any subjectivity in the assessment of the symptoms. A second limitation is the small sample size with respect to the assessment of the secondary endpoints (e.g., tearing and reddening) which may have prevented some reasonably strong associations from coming up as significant. A third limitation is the non-independence of eyes in neonates with a bilateral affection.

### Generalizability of the Trial Findings

Clinical characteristics observed in the present study were consistent with those reported in prior studies. Laterality of symptoms occurred in approximately one-third of cases, consistent with prior reports (20–23). Ocular discharge was accompanied by tearing in 63% of neonates at baseline. Tearing is described also in ~80–90% of patients with CNLDO but is not systematically present in preterm neonates because the tear production is not fully developed (20, 22–25). In general, ocular discharge can also be a first sign of CNLDO, which cannot definitively be ruled out at first stage of illness.

We found a success rate at 96 h of 53.1% which is in accordance with Stolovitch et al. who showed a success rate (defined as no epiphora or discharge) of 56% after the first attempt of lacrimal sac massage in children aged <2 months with CNLDO (26).

None of the neonates tested positive for *Neisseria gonorrhoeae* or *C. trachomatis*. This supports the efficacy of the preventive measures applied in Switzerland. No association was observed between the microbiological flora and the success of therapy. This underlines the different possible evolutions for the same pathogen and thus supports the use of conservative management as a first-line treatment. Furthermore, as preterm neonates have a premature immune system, the use of antibiotic therapy in these individuals might facilitate the overgrowth of resistant bacteria in the nasolacrimal system (22).

We observed a trend for a shorter time to reddening resolution and less relapse of reddening with Euphrasia, as well as a trend for less tearing at 96 h. This is in the line with previous studies showing promising results regarding the use of Euphrasia in the symptomatic treatment of conjunctivitis of any etiology (i.e., reddening, burning, and veiling of vision) (13).

### Conclusions and Implications

In this study, Euphrasia did not improve ocular discharge at 96 h and did not decrease the use of topical antibiotic therapy. However, this study provides evidence of the clinical efficacy of conservative therapy (i.e., lacrimal sac massage) as a first-line treatment in preterm neonates with ocular discharge. No relationship between the microbiological agent identified and the success of treatment was observed. These results support that topical antibiotics should not be used systematically as the first-line treatment of ocular discharge, including in preterm neonates. Finally, results suggest that Euphrasia may be of benefit for symptoms such as reddening and tearing, and thus improve the comfort of patients.

## Data Availability Statement

The datasets generated for this study are available on request to the corresponding author.

## Ethics Statement

The studies involving human participants were reviewed and approved by Ethics Commission of the Canton of Bern, Switzerland (215/08). Written informed consent to participate in this study was provided by the participants' legal guardian/next of kin.

## Author Contributions

UW and MN conceived the study. UW supervised the research team. LS and ME participated in designing the study. UW, SK, LS, and TK participated in operationalizing procedures, participant recruitment, project coordination, and study execution. ME and VM provided the study medication and participated in operationalizing medication procedures. GG and DM-G wrote the first draft of the manuscript. All authors participated in the revision of subsequent drafts and all approved the final version of the manuscript.

## Conflict of Interest

The authors declare that the research was conducted in the absence of any commercial or financial relationships that could be construed as a potential conflict of interest.

## Footnotes

^1^(2010). “EudraLex - The rules governing medicinal products in the european union.”
