# Supplementary material for: Euphrasia Eye Drops in Preterm Neonates With Ocular Discharge: A Randomized Double-Blind Placebo-Controlled Trial
Source: Front Pediatr. 2020 Aug 11;8:449. doi: 10.3389/fped.2020.00449 (PMC7431947; doi:10.3389/fped.2020.00449)
Supplement: Supplementary file 1 [file Data_Sheet_1.pdf]

Supplementary Material

Table E1. Ocular health at baseline according to use of topical antibiotic therapy (n=84 neonates, 111 affected eyes)

	**No topical antibiotic therapy (n=87 eyes)**	**MD**	**Topical antibiotic therapy (n=24 eyes)**	**MD**	**p-value[a]**
Ocular discharge at baseline	85 (97.7%)[b]	-	24 (100.0%)		0.99
Type of ocular discharge		1		-	0.50
White	11 (13.1%)		2 (8.3%)		
Yellow	70 (83.3%)		20 (83.3%)		
Green	3 (3.6%)		2 (8.3%)		
Reddening	42 (48.8%)	1	12 (50.0%)	-	0.92
Tearing	49 (58.3%)	3	21 (87.5%)	-	0.008

Data are expressed as number (percentage)

MD, missing data

[a] Chi-square test or Fischer test, as appropriate.

[b] Two neonates did not fill the inclusion criteria (intention-to-treat approach)

Table E2. Results of swabs performed at baseline according to treatment arm and use of topical antibiotic therapy (n=84 neonates)

	**Topical antibiotic therapy (n=16)**			**No topical antibiotic therapy (n=68)**		
	**Euphrasia (n=8 neonates)**	**Placebo (n=8 neonates)**	**p-value[a]**	**Euphrasia (n=34 neonates)**	**Placebo (n=34 neonates)**	**p-value[a]**
Positive swab	7	6	0.99	16 (47.1%)	15 (44.1%)	0.81
Microbiological agents in positive swabs						
Staphylococcus aureus	5	4		8	8	
Klebsiella pneumonia	1	0		1	0	
Pseudomonas aeruginosa	0	1		1	1	
Serratia marcescens	1	0		1	2	
Serratia marcescens + Enterobacter cloacae	0	1		0	0	
Citrobacter koseri + Staphylococcus aureus	0	0		1	0	
Corynebacterium macginleyi+ Staphylococcus aureus	0	0		1	0	
Escherichia coli	0	0		0	2	
Escherichia coli + Enterococcus faecalis	0	0		1	0	
Enterobacter cloacae	0	0		1	0	
Klebsiella oxytoca	0	0		0	1	
Moraxella catarrhalis	0	0		1	0	
Staphylococcus aureus + Pseudomonas aeruginosa	0	0		0	1	

Data are expressed as number (percentage)

[a] Chi-square test or Fischer test, as appropriate.

At inclusion, before the start of the therapy, a bacterial/viral and chlamydial conjunctival swab was conducted systematically.

Table E3. Success of treatment at 96 hours in neonates who did not receive any topical antibiotic therapy according to positive or negative swab (n=68 neonates)

	Positive swab at inclusion (n=31)					Negative swab at inclusion (n=37)				
	**Euphrasia (n=16 neonates**	**MD**	**Placebo (n=15 neonates)**	**MD**	**p-value[a]**	**Euphrasia (n=18 neonates)**	**MD**	**Placebo (n=19 neonates)**	**MD**	**p-value[a]**
No ocular discharge at 96 hours	11 (71.4%)	1	10 (66.7%)	-	0.99	12 (66.7%)	-	10 (55.6%)	1	0.38

Data are expressed as number (percentage)

[a]Chi-square test or Fischer test, as appropriate.

Success of treatment was defined as no ocular discharge at 96 hours and no use of topical antibiotic therapy during the 96-hour intervention period. If a neonate presented a bilateral affection, the treatment was defined as successful only if ocular discharge had disappeared in both eyes at 96 hours.

MD, missing data
